# An Optimized/Scale Up-Ready Protocol for Extraction of Bacterially Produced dsRNA at Good Yield and Low Costs

**DOI:** 10.3390/ijms24119266

**Published:** 2023-05-25

**Authors:** Lucas Henrique Figueiredo Prates, Maximilian Merlau, Johanna Rühl-Teichner, Marc F. Schetelig, Irina Häcker

**Affiliations:** Department of Insect Biotechnology in Plant Protection, Justus Liebig University Giessen, 35394 Giessen, Germany; maxmerlau@web.de (M.M.); johanna.ruehl@vetmed.uni-giessen.de (J.R.-T.); marc.schetelig@agrar.uni-giessen.de (M.F.S.)

**Keywords:** double-stranded RNA, bacterial production system, RNA interference, RNAi-based pest control, dsRNA production costs, RNA-based biopesticides, organic purification, liquid–liquid extraction, selective extraction

## Abstract

Double-stranded RNA (dsRNA) can trigger RNA interference (RNAi) and lead to directed silencing of specific genes. This natural defense mechanism and RNA-based products have been explored for their potential as a sustainable and ecofriendly alternative for pest control of species of agricultural importance and disease vectors. Yet, further research, development of new products and possible applications require a cost-efficient production of dsRNA. In vivo transcription of dsRNA in bacterial cells has been widely used as a versatile and inducible system for production of dsRNA combined with a purification step required to extract the dsRNA. Here, we optimized an acidic phenol-based protocol for extraction of bacterially produced dsRNA at low cost and good yield. In this protocol, bacterial cells are efficiently lysed, with no viable bacterial cells present in the downstream steps of the purification. Furthermore, we performed a comparative dsRNA quality and yield assessment of our optimized protocol and other protocols available in the literature and confirmed the cost-efficiency of our optimized protocol by comparing the cost of extraction and yields of each extraction method.

## 1. Introduction

RNA interference (RNAi) is a natural defense mechanism against foreign RNA, triggered by the presence of double-stranded RNA (dsRNA) and resulting in the silencing of specific genes via mRNA degradation or block of translation. Since its discovery in 1998 in the nematode *Caenorhabditis elegans* [1], RNAi has been transformed into a molecular technique to selectively silence gene expression and it has been extensively used in many different organisms. Besides basic and widely used gene function studies via RNAi-mediated gene knockdown and assessment of the corresponding phenotype, RNAi is also being studied for its application in gene therapy [2] or cancer therapy [3,4]. In addition, it can be used to screen lists of disease-causing gene candidates [5], just to name a few examples.

In recent years, RNAi has been applied to insect pest control, as it offers the possibility to species-specifically kill pest species by silencing essential genes. For instance, ingestion of bacterial cells expressing dsRNA targeting the heat shock protein and the shibire genes lead to gene silencing and lethality at the larval stage of the invasive emerald ash borer (*Agrilus planipennis* Fairmaire) [6]. Another possible strategy for insect pest control by RNAi is to target genes responsible for the (metabolic) resistance against commonly used insecticides [7]. Comprehensive reviews on this topic, including current achievements related to technical and insect-specific challenges, potential applications, regulatory considerations, and perspectives of RNAi for insect control have been recently published [8,9,10,11].

The potential of RNAi-based approaches for pest control has also gained attention in the industry. The first RNAi-based product for pest control to obtain approval from a regulatory agency was the maize “SmartStax^®^ Pro” (Bayer, Leverkusen, Germany) approved in 2017 in the US [12]. According to its manufacturer, it expresses dsRNA that interferes in the natural production of a vital protein in the corn rootworm. Therefore, ingestion of this maize would be lethal for the insect. Other companies, such as Syngenta, also announced advances in the development of RNAi-based products (https://www.syngenta.com/en/innovation-agriculture/research-and-development/rna-based-biocontrols, accessed on 24 April 2023).

The application of RNAi for selective gene silencing experiments in the laboratory requires the production of gene-specific dsRNA, which can be obtained through different approaches, including in vitro transcription or in vivo production using genetically modified bacteria or yeast strains [13,14,15]. While the production of microgram to one digit milligram amounts of dsRNA via in vitro transcription is typically not a problem for the budget, the production of higher milligram or even gram amounts of clean dsRNA, for large scale laboratory experiments for insect pest control, can become financially challenging [13,16,17]. Here, the production of dsRNA via genetically modified bacterial cells can be an alternative.

Since its genetic modification to silence expression of RNase III and the insertion of a T7- inducible system for production of dsRNA [18,19], the bacterial strain HT115 (DE3) has been widely used for dsRNA production [6,19,20,21,22,23]. Although the application of bacterial crude extracts containing the produced dsRNA has been investigated in gene silencing experiments [19,20,22,24,25,26], purified dsRNA, free of other bacterial nucleic acids and proteins, is preferable or required in many cases, as it not only avoids potential immune responses due to the presence of bacterial cells [27], but also facilitates regulatory approvals [10,28].

Several methods have been described for dsRNA extraction from bacterial cells [29,30,31,32,33,34]. Phenol–guanidine-based protocols have been reported as efficient method for extraction of dsRNA at high yields (up to 30 µg/mL of bacterial cells) and good quality [29,34,35]. Other alternatives rely on fixing bacterial cells in ethanol and phosphate-buffered saline solution for extraction of dsRNA [32,33] or performing a phenol/chloroform/isoamyl alcohol (P/C/I) extraction and further DNase and RNase digestions [30,31].

The purification based on commercially available phenol–guanidine solutions inflates dsRNA production costs for medium- to large-scale laboratory experiments. Scaling up published protocols to extract dsRNA for example from a 5 L-bacterial culture, e.g., following the protocol from Verdonckt et al.’s work [34], would consume 500 mL of QIAzol^®^ (QIAGEN, Venlo, The Netherlands), corresponding to 771.75 EUR just for the organic reagent. Processing the same amount of cells with a protocol using TRIzol^TM^ (Invitrogen, Waltham, MA, USA) [29] would cost nearly EUR 1700 (794 mL of the organic reagent needed). While the required amounts of dsRNA for effective gene silencing vary with the target species, it is predicted that 2 to 10 g of dsRNA per hectare for crop protection would be needed in the field [36]. Based on the published yields, this would correspond to EUR 30,000 to 142,000 for TRIzol^TM^, or EUR 38,000 to 192,000 for QIAzol^®^. However, for medium- to large-scale laboratory experiments the costs would also quickly be in the thousands to tens of thousands of Euros.

While the price for dsRNA synthesis has dropped strongly nowadays, with predicted production costs as low as 1 USD/g for a proprietary cell-free methodology (www.greenlightbiosciences.com, accessed on 24 April 2023), the versality of engineered HT115 (DE3) cells for dsRNA production allows scientists to readily adjust dsRNA sequences according to experimental requirements or for distinct applications. Having a reliable and cost-effective protocol for extraction of dsRNA from large HT115 (DE3) culture volumes would further broaden the usability of HT115 cultures for dsRNA production, e.g., for field trials or larger laboratory applications, such as high-throughput screening of potential targets for RNAi in different species. Therefore, we aimed to develop a low-cost protocol for dsRNA extraction with high yield and quality, but at a fraction of the costs from the best currently available alternatives.

## 2. Results

### 2.1. dsRNA Can Be Successfully Extracted from HT115 DE3 Cells Using Either TRIzol^TM^ or P/C/I

**Figure 1:**
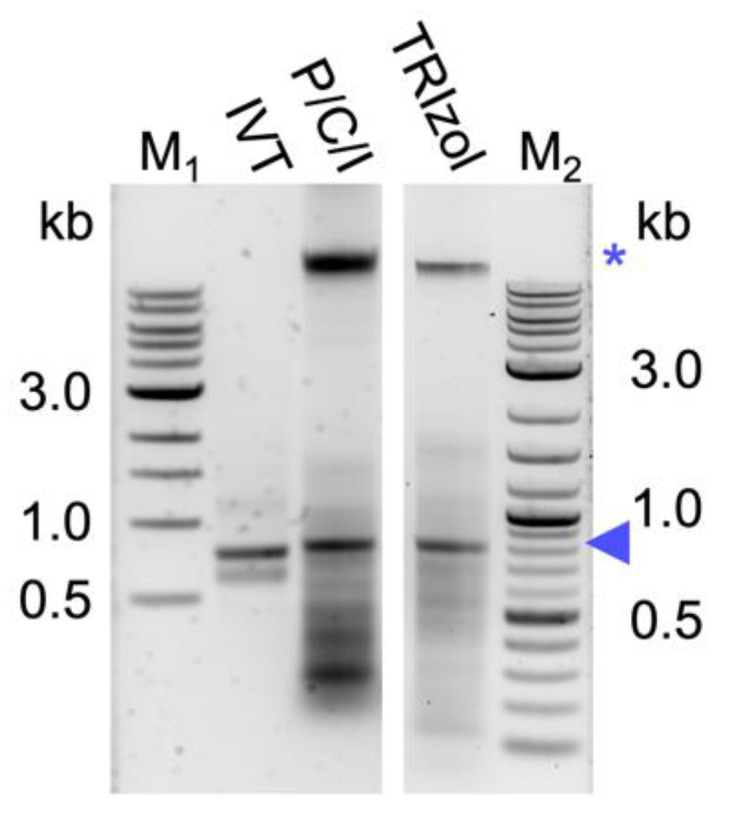
Comparison of yield and quality of bacterially produced dsRNA extracted with the protocol established by Ongvarrasopone et al. [29] using either P/C/I pH 4.5-5 or TRIzol^TM^. In vitro transcribed (IVT) dsRNA (0.4 µg) is shown as a reference. Loaded were 2.8 µg and 1.5 µg of dsRNA extracted using P/C/I or TRIzol^TM^, respectively. Blue arrowhead indicates the band of the dsRNA; the asterisk indicates a high-molecular-weight band, presumably genomic DNA, copurified from the bacterial cells. M1: 1 kb ladder. M2: 1 kb plus ladder (New England BioLabs Inc., Ipswich, MA, USA).

Several protocols for dsRNA extraction from bacterial cells have been published [29,30,31,32,33,34]. A protocol that reported good yield (30 µg per OD_600_ of cells) and purity of dsRNA uses boiling of the cells in sodium dodecyl sulfate (SDS), followed by RNase A digest of bacterial RNAs and TRIzol^TM^ extraction of the dsRNA [29]. Applying TRIzol^TM^ extraction to larger scale batch cultures or small fermenters, however, produces considerable costs, as described above. We therefore evaluated dsRNA extraction from HT115 DE3 cells induced to produce dsRNA at OD_600_ 0.8 following the general workflow of the phenol–guanidine-based protocol [29], but replacing the TRIzol^TM^ with the cheaper chemical P/C/I (25:24:1, pH 4.5–5) and modifications to buffer to cell volume ratio and RNase A incubation time. The corresponding in vitro transcribed dsRNA was used as reference in gel electrophoresis. The obtained bands of the expected size indicate successful extraction of bacterially produced dsRNA using both TRIzol^TM^ and P/C/I (25:24:1, pH 4.5–5) (Figure 1). Spectrophotometric quantification of the nucleic acids at 260 nm showed comparable mass yields per OD cells with both extraction reagents. However, the relative amount of a copurified high-molecular-weight band (presumably bacterial genomic DNA (gDNA)) and smaller bacterial RNAs relative to the dsRNA band was higher when using P/C/I for dsRNA extraction. We therefore started to systematically assess the nature of the copurified high-molecular-weight band, the influence of buffer pH, serial P/C/I extractions, and induction OD_600_ on the quality and yield of P/C/I-extracted dsRNA. Moreover, with regard to applying the protocol to dsRNA extractions from liters of culture, we assessed the efficiency of cell lysis in the reduced lysis buffer volumes, which reduces the amount of P/C/I needed for extraction.

### 2.2. DNase Digestion Confirms Presence of Bacterial Genomic DNA in Extracted dsRNA

**Figure 2:**
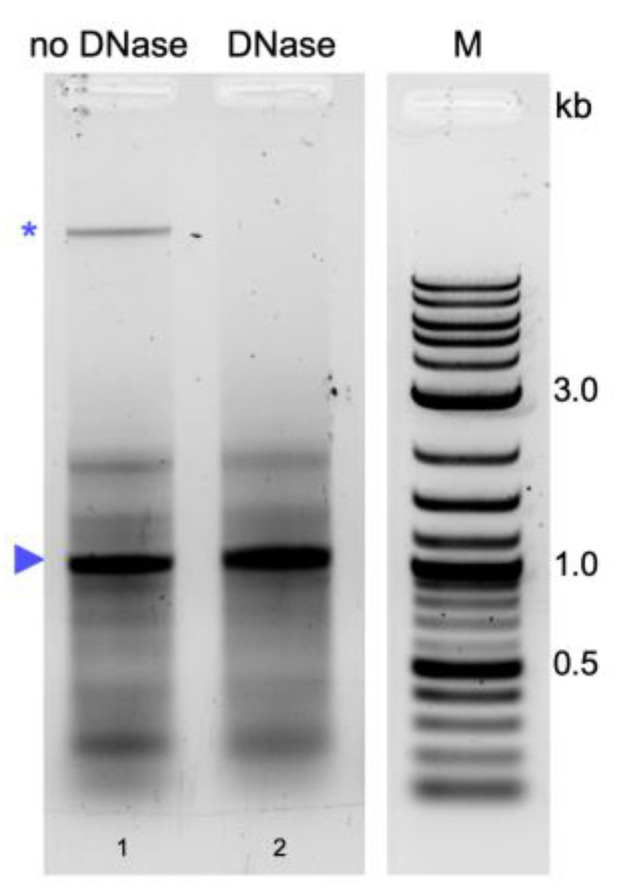
Effect of DNase treatment on dsRNA purity. Shown are the products of phenol–guanidine-based extraction following the protocol established by Ongvarrasopone et al. [29] (same conditions as in Figure 1) with mock DNase digestion (lane 1, no DNase) or with DNase digestion (lanes 2, DNase). Loadings were of 1.5 µg for each sample. Blue arrowhead indicates extracted dsRNA, blue asterisk indicates bacterial DNA. M: 1 kb plus ladder (New England BioLabs Inc., Ipswich, MA, USA).

TRIzol^TM^ as well as P/C/I extraction resulted in copurification of a high-molecular-weight nucleic acid. A similar high-molecular-weight band was observed in protocols without DNase treatment of the purified dsRNA [20,29,32], but was absent in protocols applying a DNase digestion [30,31]. We therefore included a DNase treatment step in the phenol–guanidine-based protocol [29]. This treatment completely removed the high-molecular-weight band compared to the extraction without DNase treatment (Figure 2, lane 2 compared to lane 1), showing that it consisted of bacterial (genomic) DNA.

### 2.3. Boiling in the Presence of Anionic Surfactant Efficiently Opens Bacterial Cells for dsRNA Extraction

**Figure 3:**
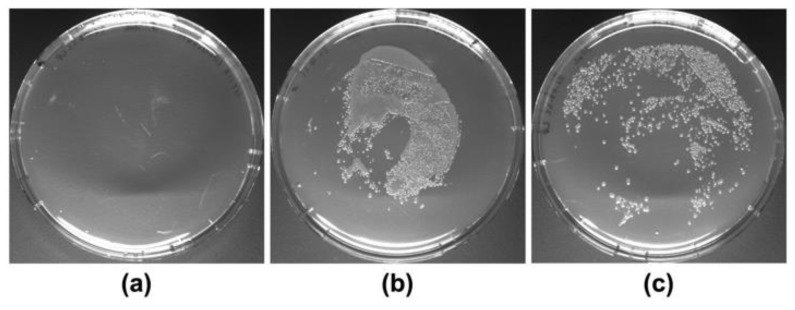
Cell viability assay with HT115 DE3 cells resuspended in reduced volume of 0.1% (*w*/*v*) SDS and boiled at 95 °C for 2 min (**a**) compared to cells resuspended in 2% (*w*/*v*) LB-medium without boiling and incubated at room temperature (**b**,**c**). Plated were 1 µL of undiluted cell suspension in (**a**,**b**), and 10 µL of 1:100 dilution in (**c**).

Efficient cell lysis is critical for efficient dsRNA extraction, as dsRNA from unlysed cells would be lost in the pellet at the P/C/I extraction step. Moreover, the volume of buffers used in the initial steps of the protocol will eventually determine the volume of organic reagent (TRIzol^TM^, QIAzol^®^ or P/C/I) required for extraction of dsRNA, thereby directly impacting the costs and suitability of any extraction protocol for larger applications. We, therefore, investigated whether reducing the volume of SDS lysis buffer per OD_600_ of cells affects cell lysis. After boiling the bacterial cells at 95 °C for 2 min with a four-fold reduced volume of 0.1% (*w*/*v*) SDS per OD_600_ of cells compared to the original protocol [29], no viable cells were observed in a cell viability assay (Figure 3). Thus, boiling in the reduced volume of 0.1% SDS efficiently opens up bacterial cells for dsRNA extraction.

### 2.4. Low pH in a Second P/C/I Extraction Reduces Amount of Copurified DNA

**Figure 4:**
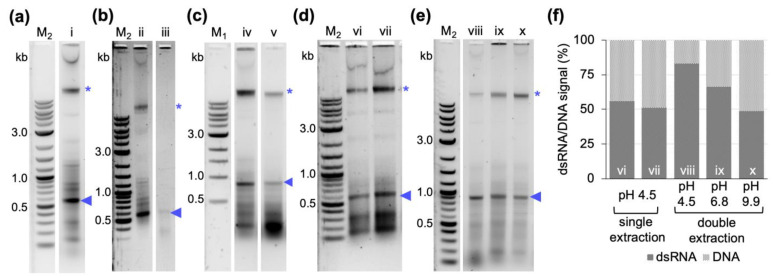
Comparison of different purification conditions on dsRNA purity and yield. (**a**) Product of single P/C/I extraction from cells lysed in 0.1% (*w*/*v*) SDS pH 7.1 and single-stranded RNA degradation performed in RNase A buffer at pH 7.5 (i) following the protocol established by Ongvarrasopone et al. [29] (same conditions as in Figure 1) (**b**) Cell lysis performed in 0.1% (*w*/*v*) SDS pH 7.1 (ii) or in 0.1% (*w*/*v*) SDS and 0.1 M sodium acetate buffer pH 4.5 (iii). (**c**) Single-stranded RNA degradation performed in RNase A buffer at pH 7.5 (iv) or pH 4.5 (v). (**d**) Reaction was acidified with 0.1 M sodium acetate buffer pH 4.5 before addition of P/C/I (vi and vii). (**e**) Product of the first P/C/I extraction was adjusted to pH 4.5, 6.8, or 9.9 (lanes (viii), (xi), and (x), respectively) by adding the respective buffers and a 2^nd^ P/C/I extraction was performed. (**f**) Relative integrated densities of dsRNA and DNA bands in samples from (**d**,**e**) quantified with Adobe Photoshop (Adobe Inc., 2023, San José, CA, USA). Displayed is the percentage of the respective band intensities compared to the sum of dsRNA and DNA densities per lane. Loading amounts were 2.6 µg in (**a**), 3 µg in (**b**), 2.8 µg in (**c**), 2.0 µg in (**d**) and 1.1 µg in (**e**). Blue arrowheads indicate extracted dsRNAs of sizes 800 or 480 bp, blue asterisks indicate bacterial DNA copurified from the bacterial cells. M1: 1 kb ladder. M2: 1 kb plus ladder (both New England BioLabs Inc., Ipswich, MA, USA).

DNase treatment of the extracted dsRNA might not be applicable to the processing of large culture batches. We therefore focused on the chemical approach of separating RNA and DNA to provide an alternative option of removing the contaminating DNA. At slightly alkaline conditions (pH 7.5–8.0), RNA and DNA partition into the aqueous phase of a phenol/chloroform extraction after phase separation, while at pH 4–6, the DNA should be retained in the lower, i.e., organic, phase and interphase [35]. Therefore, P/C/I pH 4.5–5.0 was chosen in our protocol for dsRNA extraction. Nevertheless, bacterial DNA was copurified (Figure 4a). To test if this contaminating DNA could be removed by further acidifying the extraction conditions, we reduced the pH of the RNase A buffer or the SDS 0.1% (*w*/*v*) lysis buffer to pH 4.5 before performing the extraction with P/C/I pH 4.5–5.0.

However, these treatments not only yielded weaker dsRNA bands, but also increased the contamination with small co-extracted RNAs, suggesting that low-pH conditions reduced RNase A activity (Figure 4b,c, compared to product of P/C/I extraction with unchanged cell lysis and RNase A buffers in Figure 4a). We subsequently assessed whether acidifying the reaction after RNase A digest and before P/C/I addition by adding 2.5 sample volumes of 0.1 M sodium acetate buffer, pH 4.5 would remove the DNA band (Figure 4d). However, this did not improve the dsRNA purity compared to the P/C/I extraction without additional acidification. It again increased the amount of copurified bacterial RNAs. In the next step we, therefore, included a 2^nd^ acidic P/C/I extraction step into the protocol. For this, the dsRNA solution obtained after the first P/C/I extraction and nucleic acid precipitation was acidified by adding three sample volumes of 0.1 M sodium acetate buffer, pH 4.5, followed by a 2^nd^ P/C/I extraction and nucleic acid precipitation. This treatment yielded strongly improved dsRNA purity compared to the result of a single P/C/I extraction (Figure 4e, lane viii, compared to Figure 4a, lane i). Two control experiments applying a 2^nd^ P/C/I extraction at neutral (pH 6.8) or basic conditions (pH 9.9) (Figure 4e, lanes ix and x) showed that the combination of 2^nd^ P/C/I extraction and low pH is needed for the effect. The improved dsRNA to DNA band ratio observed via gel electrophoretic analysis was confirmed by quantifying the integrated density of the bands with Adobe Photoshop (Adobe Inc., 2023, San José, CA, USA). The relative density of the dsRNA to DNA band is considerably higher when a second P/C/I extraction is performed with intermediate acidification of the sample (Figure 4f).

### 2.5. Inducing dsRNA Production at a Late Exponential Phase Reduces Yield and Quality of Extracted dsRNA

**Figure 5:**
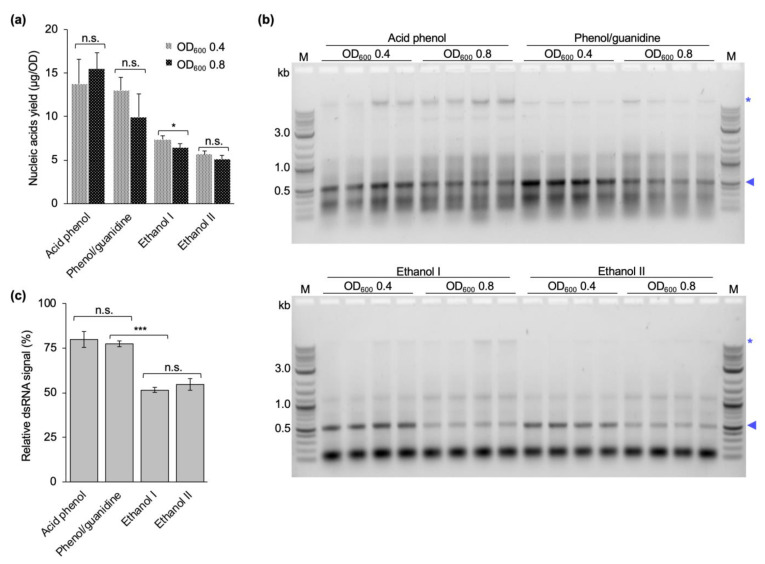
Yield and purity of dsRNA extracted from bacterial cells induced to produce dsRNA at OD_600_ of 0.4 or 0.8. Comparative extractions were performed with the acid phenol-based protocol developed in this study, the phenol–guanidine-based protocol established by Ongvarrasopone et al. [29] (Phenol/guanidine), or the ethanol-based protocols established by Posiri et al. [32] (Ethanol I) or Papić et al. [33] (Ethanol II). (**a**) Nucleic acid yield (µg per 1 OD_600_ of cells) extracted from bacterial cells induced to produce dsRNA at OD_600_ of 0.4 (grey) or 0.8 (black), measured by spectrophotometry at 260 nm. Data shown here are based on two technical replicates (extractions) from two biological replicates. n.s.: no statistically significant difference. *: statistically significant difference, *p*-value < 0.05. (**b**) Gel electrophoresis of dsRNA extracted from bacterial cells induced to produce dsRNA at OD_600_ of 0.4 or 0.8. Shown are the results of two technical replicates from two biological replicates for each condition (i.e., dsRNA was extracted from two samples per culture, for two different cultures). In all lanes, the equivalent of 0.2 OD_600_ of cells was loaded. Blue arrowheads indicate extracted dsRNA, blue asterisks indicate bacterial DNA. M: 1 kb plus ladder (New England BioLabs Inc., Ipswich, MA, USA). (**c**) Percentage of dsRNA obtained from cultures induced at OD_600_ of 0.8 compared to induction at OD_600_ of 0.4. Integrated density of dsRNA bands from the gels shown in (**b**) was quantified with Adobe Photoshop (Adobe Inc., 2023, San José, CA, USA), the intensity of the band obtained from the lower induction OD_600_ set to 100% and the intensity of the high induction OD_600_ put in relation; n.s. = no statistically significant difference. *** statistically significant difference, *p*-value < 0.001 (one-way ANOVA, Holm–Sidak method for all pairwise multiple comparison).

In vivo transcription of dsRNA in HT115 DE3 cells transformed with the vector L4440 can be induced with IPTG at any stage of bacterial growth. Considering different published manuscripts that induce dsRNA production in bacterial cells between OD_600_ of 0.4 and 0.8, we evaluated whether induction with IPTG during a late exponential phase of the bacterial growth would result in higher yields of dsRNA per bacterial cell. Thus, the dsRNA production was induced at an OD_600_ of either 0.4 or 0.8, and dsRNA purified with published protocols [29,32,33] and our own optimized protocol including the 2^nd^ acidic P/C/I extraction. Nucleic acid yields determined via absorption measurement at 260 nm (A_260_) of the purified nucleic acids did not significantly (α = 0.05) increase when cultures were induced at a higher OD_600_ for three of the four protocols tested. For the ethanol-based protocol established by Posiri et al. [32], the induction at late growth stage even yielded significantly less nucleic acids per OD_600_ of cells (*p* = 0.028) (Figure 5a). Quality assessment of the purified nucleic acids via gel electrophoresis, however, revealed clear differences in the quality and quantity of the purified dsRNA at different induction ODs for all tested protocols. First, the band corresponding to the dsRNA was markedly stronger in relation to the background of copurified nucleic acids when induction of dsRNA production occurred at OD_600_ of 0.4. Second, the absolute dsRNA band intensity from cells induced at OD_600_ of 0.4 was stronger compared to the induction at OD_600_ of 0.8 (Figure 5b, same equivalent of cells was loaded). This was confirmed by quantifying the integrated densities of the dsRNA bands and of the complete lane by Adobe Photoshop to determine the fraction of dsRNA within each lane. Setting the fraction of dsRNA obtained from induction at OD_600_ 0.4 to 100 % for each purification protocol showed that induction at OD_600_ 0.8 results in 20.2 to 48.5 % less dsRNA per OD of cells (Figure 5c). Thus, induction at higher OD_600_ does not improve the amount of dsRNA produced per bacterial cell, nor the purity of the extracted dsRNA.

### 2.6. Comparative Assessment of Yield and Purity of the Optimized Low-Cost Protocol

**Figure 6:**
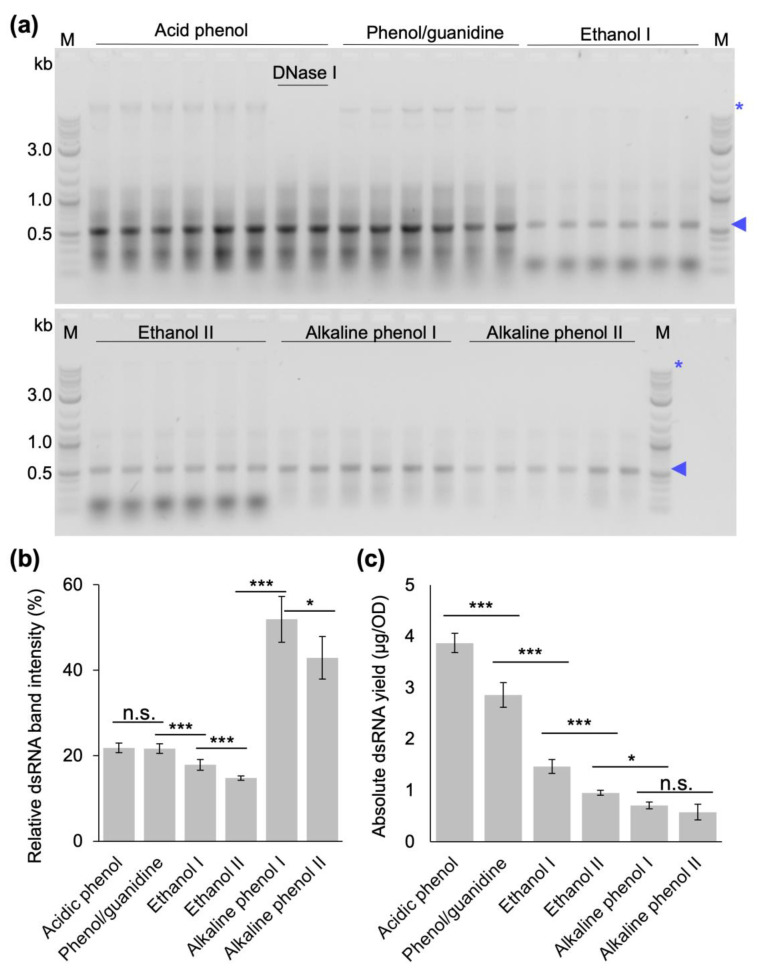
Comparative gel electrophoretic analysis, relative and absolute yield of dsRNA extracted from bacterial cells with different extraction protocols. Extractions were performed from cells induced at OD_600_ 0.4 with the acid phenol-based protocol developed in this study, the phenol–guanidine-based protocol established by Ongvarrasopone et al. [29] (Phenol/guanidine), the ethanol-based protocols established by Posiri et al. [32] (Ethanol I) or Papić et al. [33] (Ethanol II), the alkaline phenol-based protocols established by Ahn et al. [31] (Alkaline phenol I) or Solis et al. [30] (Alkaline phenol II). (**a**) Agarose gel electrophoresis of extracted dsRNA. Shown are the results of two technical replicates (i.e., extractions) from three biological replicates (i.e., bacterial cultures) for each extraction protocol. The product of the acid phenol-based protocol developed in this study with an additional DNase I digestion for one of the biological replicates is also shown. In all lanes, the equivalent of 0.4 OD of cells was loaded. (**b**) Relative dsRNA amount per lane (integrated density of the dsRNA band compared to the integrated density of the whole lane quantified by Adobe Photoshop (Adobe Inc., 2023, San José, CA, USA)). Data are based on gel shown in (**a**). (**c**) Absolute amount of dsRNA obtained from one OD of cells, calculated from the spectrophotometric analysis (absorbance at 260 nm) and the relative band intensities in (**b**) for each of the protocols tested. n.s. = no statistically significant difference. * statistically significant difference, *p*-value < 0.05, *** statistically significant difference, *p*-value < 0.001 (one-way ANOVA, Holm–Sidak method for all pairwise multiple comparison). Blue arrowheads indicate extracted dsRNA, blue asterisks indicate bacterial DNA. M: 1 kb plus ladder (New England BioLabs Inc., Ipswich, MA, USA).

We finally conducted a comparative dsRNA quality and yield assessment of our optimized P/C/I extraction protocol and the published protocols by performing dsRNA extractions from the same culture batches for all protocols in three biological replicates and two technical replicates each. To confirm that the remaining traces of the high-molecular-weight band copurified in our optimized protocol is DNA, as observed for the extraction with TRIzol^TM^ (Figure 2), we additionally included a facultative DNase I treatment step in the protocol before acidifying the sample for the second P/C/I extraction in one of the biological replicates (Figure 6a). In this comparative experiment, our protocol resulted in significantly higher nucleic acid amounts as determined by spectrophotometric analysis (17.7 ± 1.24 µg/OD_600_) than even the best published protocol established by Ongvarrasopone et al. [29] (13.2 ± 1.00 µg/OD_600_, *p*-value < 0.001), and two- to ten-fold higher nucleic acid amounts than both versions of the ethanol-based protocol [32,33] (8.2 ± 0.31 and 6.5 ± 0.22 µg/OD_600_, respectively (*p*-value < 0.001)) and the alkaline phenol-based protocols [30,31] (1.4 ± 0.14 and 1.4 ± 0.42 µg/OD_600_, respectively (*p*-value < 0.001)). DsRNA purity assessment via gel electrophoresis showed some background of different-size copurified bacterial nucleic acids in our protocol and the phenol–guanidine-based protocol established by Ongvarrasopone. et al. [29]. Both ethanol-based protocol presented a strong co-extraction of small bacterial RNAs; and both alkaline phenol-based protocols with RNase A and DNase I digestions produced overall the cleanest dsRNA, but at very low quantitative yields (Figure 6a). Considering that the absorbance measurements used to quantify the yields do not distinguish between the desired dsRNA and co-extracted bacterial nucleic acids in a sample, we determined the relative dsRNA amount per sample with Adobe Photoshop (integrated band densities, Figure 6b) and with these numbers calculated the absolute amount of dsRNA per sample (Figure 6c). This analysis showed that the highest absolute amount of dsRNA per OD cells can be extracted with our optimized protocol, followed by the protocol of Ongvarrasopone et al. Three- to five-fold lower absolute yields of dsRNA were obtained using the ethanol-based and alkaline phenol-based protocols.

### 2.7. P/C/I Extraction of Bacterially Produced dsRNA Is Cost-Effective

A cost analysis of the extraction protocols considering only the most expensive part, the organic reagent, resulted in estimated costs of EUR 3.58 to process 400 mL-bacterial culture with our optimized protocol. On the other hand, using either QIAzol^®^ or TRIzol^TM^ increases the dsRNA extraction costs to EUR 60 or even more than 100, respectively, when scaling the respective protocols up to 400 mL culture, while the ethanol-based protocols cost less than EUR 1 for this culture volume. A more realistic cost analysis considers the costs for obtaining a specific dsRNA amount. Based on the absolute dsRNA yields determined in our comparative experiment for each of the protocols (Figure 6c), we therefore calculated the costs to obtain one milligram of dsRNA. Compared to our optimized protocol, the ethanol-based protocol [32] is approximately 31% cheaper in producing the same amount of dsRNA. This calculation, however, does not yet include the costs for media preparation, which would be about four times higher for the ethanol-based protocols. On the other hand, our optimized protocol is approximately 90 to 98% cheaper than using the alkaline phenol-based protocols or the phenol–guanidine-based version that uses TRIzol^TM^, respectively, to produce the same amount of purified dsRNA.

## 3. Discussion

Several methods have been developed for extraction of bacterially produced dsRNA for application of RNAi in crop protection, vector control, or functional genomic analysis, among other research fields [29,30,31,32,33,34,37]. In the initial experiments, we had tested most of these protocols for the dsRNA yield and purity and obtained the best results in yield with the protocol established by Ongvarrasopone et al., which is based on a TRIzol^TM^ extraction. TRIzol^TM^ (Invitrogen), TRI Reagent^®^ (Merck, Darmstadt, Germany) and QIAzol^®^ (QIAGEN) are examples of commercially available phenol–guanidine-based reagents that have been used for extraction of bacterially produced dsRNA for RNAi application [29,34]. They offer an all-in-one solution for cell lysis, protein denaturation and deactivation of nucleases by the chaotropic agent guanidinium thiocyanate in solution with phenol [35]. However, these commercially available monophasic solutions of phenol–guanidine are expensive. For example, in a protocol using QIAzol^®^ the costs for the reagent contribute to up to 85% of the total costs of dsRNA extraction [34]. This becomes especially relevant when upscaling dsRNA purification from several liters of culture. Therefore, we exchanged the TRIzol^TM^ for the about 10-fold cheaper chemical P/C/I (25:24:1), pH 4.5–5. This resulted in a higher background of copurified bacterial RNAs and also (genomic) DNA (Figure 1). The latter was unexpected, as the acidic conditions of the P/C/I extraction should partition the DNA into the interphase and organic phase [35]. However, the acidity of the P/C/I itself was not sufficient for this effect, and also acidifying the reaction before the P/C/I addition did not improve the partitioning. In a systematic optimization process we found that adding a 2^nd^ acidic P/C/I extraction step reduces the copurified nucleic acids to a similar level as in the original TRIzol^TM^-based protocol. Serial extractions to improve the quality of the dsRNA have previously been performed not only with phenol/chloroform-based protocols [30,31] but also with phenol–guanidine-based extraction methods [34]. Altogether, our findings suggest that the pH of the used P/C/I might not always be sufficient to result in effective separation of RNA and DNA in phenol-based extractions, and that stabilization of the acidic conditions in the sample by using a low pH-buffer might be needed to obtain better results. Moreover, the presence of certain chemicals in the aqueous phase might interfere with the proper separation of the nucleic acids in the respective phase during the P/C/I extraction, as in our case the additional acidification of the reaction before adding P/C/I did not improve the result. Only when the already one-step P/C/I-purified dsRNA dissolved in pure water was acidified, followed by a 2^nd^ P/C/I extraction, could we achieve a better DNA and RNA separation. We suspect the SDS of the lysis buffer to be the interfering substance.

With regard to reducing the overall dsRNA production costs, we looked into possibilities to further increase the yield per culture volume and reduce extraction volumes to save on the most expensive component of the protocol, the organic reagent. 

Cell lysis is a critical step in the extraction of bacterially produced dsRNA, as dsRNA would be lost with unlysed cells. Pre-treatment of cells, including sonication, heating, and enzymatic digestions, has been shown to increase the yield of dsRNA extraction [31,34]. However, quality analysis via gel electrophoresis has also shown that dsRNA extracted from sonicated samples yields less strong bands and smearing in the background, suggesting degradation of dsRNA [31,34]. We could show here that boiling in a reduced volume of the published buffer [29] efficiently lysed the bacterial cells while at the same time preserving the dsRNA quality (Figure 1 and Figure 2), thereby abolishing the need for further treatments for cell lysis. Obtaining dsRNA free of bacterial cells is not only important to avoid possible unwanted immune responses in in vivo applications [27], but is also highly relevant for regulatory aspects. In Europe, for instance, if no genetically modified organism (GMO) has been used or it is proven to be inactivated, RNAi-based products intended for agricultural pest control would be regulated following the same regulations applied for classical synthetic chemical pesticides [28], which would apply to the dsRNA produced with our protocol.

Different studies in the literature have induced dsRNA production at cell densities ranging from 0.4 to 0.8 OD_600_, and harvested cells after 4 to 5 h of dsRNA production or when the OD_600_ reached 1.0 [29,30,31,32,33,34]. Following the rationale that more cells per culture volume should yield more dsRNA per culture volume, we initially induced dsRNA production at a late exponential phase (OD_600_ = 0.8). To prove this point, we additionally performed dsRNA extractions from cultures induced closer to the mid-exponential phase (OD_600_ of 0.4). Regardless of the induction timepoint, the cells were allowed to produce dsRNA for 4 h before harvesting. In contrast to our expectations, the induction at the higher OD did not yield more dsRNA. On the contrary, the amount of dsRNA produced per cell was lower. In addition, a stronger background of copurified nucleic acids was observed. This was independent of the purification protocol used (Figure 5). We assume that at a late timepoint in the exponential phase the bacterial cells are less transcriptionally active due to nutrients starting to become limiting, and maybe also become less responsive to IPTG.

Finally, we compared the yield and quality of dsRNA extracted from bacterial cells induced at OD_600_ of 0.4 with our optimized protocol, the original protocol established by Ongvarrasopone et al. and the published extraction protocols that do not require expensive monophasic solutions of phenol–guanidine [29,30,31,32,33] in extensive side-by-side extractions. This experiment showed that our optimized protocol produces the highest absolute amount of dsRNA (Figure 6), and had the highest nucleic acid concentrations. The gel electrophoretic analysis also showed that the different protocols resulted in very different dsRNA qualities. For our protocol and the phenol–guanidine-based protocol, there are still remnants of bacterial RNAs and DNA (the latter can be removed via additional DNase digestion after the first P/C/I extraction if required for specific applications). The ethanol-based protocols showed a strong contamination with small bacterial RNAs in addition to the rather moderate dsRNA yield, while, in our hands, the alkaline phenol-based protocol performed best with regard to purity, but at the lowest quantitative yields (Figure 6a,b).

Interestingly, despite following the protocols as closely as the details reported in the respective methods sections allowed, we could not reproduce the reported dsRNA yields published in the literature. Possible reasons for the differences could be the different expression systems (strains and plasmids), culture media and types (batch or fed-batch), how the yields were determined, and the dsRNA constructs themselves. For instance, it has been shown that the dsRNA construct has a significative impact on the yield of the extracted dsRNA using a phenol–guanidine-based protocol [34]. Regarding the different plasmids for expression of dsRNA, the plasmid L4440 has been used in our experiments, while pET3a and pET17b were used in some of the other studies [29,32]. Moreover, the nutrient content of the medium used to grow the cells might influence the dsRNA yield. We have preliminary data confirming that the amount of extracted dsRNA (by absorption measurement) doubles when cells are grown in the nutrient-rich terrific broth (TB) medium compared to LB medium. A very similar observation was made by Thammasorn et al. [38]. Ongvarrasopone et al., report obtaining 30 µg dsRNA per 1 OD of cells grown in 2xYT medium, while we obtained about 13 µg per OD from cells grown in LB medium with their protocol (both numbers based on absorption measurement at 260 nm, i.e., measuring the total nucleic acid content). Potentially, the combination of different expression plasmid and nutrient supply is responsible for the observed difference. Likewise, Ahn et al. obtained approximately 4.85 µg per OD from cell grown in 2xYT medium, while we obtained about 1.4 µg/OD in LB. Besides the nutrient content, the high salt present in some media, such as TB, could contribute to regulate the pH of the cultures, thereby optimizing cell growth and dsRNA production [38].

Taking into account only the costs for the organic reagent per microgram of produced dsRNA, our protocol is comparable to the two ethanol-based protocols (Table 1). All three are 15- to 75-fold cheaper than the other tested methods. When users have to decide between the three most cost-effective protocols, aspects to consider will be the use of ethanol versus P/C/I, the strong contamination with small bacterial RNAs in the ethanol-based protocols, and, last but not least, that for ethanol-based protocols about 4 times larger culture volumes are required to produce the same absolute amount of dsRNA as with our protocol. This might become a deciding factor especially for large-scale dsRNA productions.

In summary, based on the results reported in the literature and our findings with the parallel purifications, we suggest that for a true comparison of the efficiency of a purification protocol, exactly the same dsRNA production system has to be used. Moreover, besides the extraction method, also the expression system, the culture conditions, and the induction time point for dsRNA production should be taken into account to further optimize the dsRNA yields from bacterial cultures. Finally, the combination of obtained dsRNA yields, dsRNA purity, and estimated production costs per milligram of purified dsRNA (based only on the organic reagent costs) suggests that when high purity dsRNA is required for the planned experiments, the alkaline phenol-based protocols perform best, but at considerable costs if high amounts are needed. In contrast, our optimized protocol has the best combined yield to cost and yield to culture volume ratios at only a moderate copurification of bacterial nucleic acids. This makes it the ideal protocol for dsRNA production for large scale laboratory or even field trial experiments not requiring the highest purity of dsRNA, meeting also potential regulatory demands for applications in the field.

## 4. Materials and Methods

### 4.1. In Vitro Production of dsRNA

Templates for in vitro transcription (IVT) of dsRNA were produced via polymerase chain reaction (PCR) using a fragment of the gene AAEL002851 from *Aedes aegypti* cloned into pCR4 TOPO vector and primers containing at T7 promoter overhang (marked in bold). PCR reactions contained 32.6 µL of nuclease-free water, 10 µL of 5x Q5 buffer, 5 µL of 2 mM dNTP mix, 0.5 µL of forward primer P884 (**CCCTTTAATACGACTCACTATAGGG**AGAAGGAAATCATCTCCGACGAAC) at 10 µM, 0.5 µL of reverse primer P885 (**CCCTTTAATACGACTCACTATAGGG**AGAACACGGTACTGTTGCGATCC) at 10 µM, 1 ng of DNA template and 0.5 µL of Q5 Taq polymerase (New England Biolabs, Inc., Ipswich, MA, USA). The PCR reaction was performed as follows: 30 s at 98 °C; 30 cycles of 98 °C for 10 s, 65 °C for 20 s and 72 °C for 1 min; and final elongation at 72 °C for 2 min. The PCR product was analyzed and purified via gel electrophoresis using Zymo Research Gel DNA recovery kit (Zymo Research Europe GmbH, Freiburg, Germany) following the manufacturer’s instructions. The purified template (200 ng) was used to generate dsRNA via in vitro transcription using the MegaScript Kit followed by purification of the in vitro transcription product using the MEGAclear^TM^ kit according to the manufacturer’s instructions (Ambion/Life Technologies, Thermo Fischer Scientific Inc., Waltham, MA, USA).

### 4.2. In Vivo Production of dsRNA

For in vivo production of dsRNAs, 275 mL of lysogeny broth (LB-Lennox) medium (Carl Roth GmbH + Co. KG, Karlsruhe, Germany) supplemented with 100 µg/mL ampicillin (Carl Roth GmbH + Co. KG, Karlsruhe, Germany) and 12.5 µg/mL tetracycline (Fisher BioReagents, Geel, Belgium) were inoculated with 2.2 mL of an overnight culture of HT115 DE3 cells (F-, mcrA, mcrB, IN(rrnD-rrnE)1, rnc14::Tn10(DE3 lysogen: lavUV5 promoter -T7 polymerase, from the Caenorhabditis Genetics Center) transformed with L4440 plasmid encoding either a dsRNA against *eGFP* (480 bp) or *Aae beta-tubulin* (AAEL002851) (800 bp). Cells were grown at 37 °C and 180 rpm until the optical density measured at 600 nm (OD_600_) reached 0.4 or 0.8. dsRNA production was then induced via addition of isopropyl-β-D-thiogalactopyranoside (IPTG) (Carl Roth GmbH + Co. KG, Karlsruhe, Germany) to a final concentration of 0.4 mM and cells grown for another 4 h. Cells were harvested at 4 °C for 15 min at 3214 rcf, resuspended in clean LB-Lennox medium, aliquoted in batches of 10 OD or 2 OD in 2 mL-tubes, collected via centrifugation and stored at −80 °C until further use.

### 4.3. dsRNA Extraction from HT115 DE3 Cells via Phenol/Chloroform/Isoamyl Alcohol Extraction

Extraction of dsRNA using P/C/I was performed with the following modifications from the published phenol–guanidine-based protocol [29]: bacterial cells were resuspended in 12.5 µL of 0.1% sodium dodecyl sulfate (SDS) per one OD of cells and incubated for 2 min at 95 °C. Then, 16.25 µL of neutral (pH 7.5) solution of 300 mM sodium acetate, 10 mM Tris-HCl, 5 mM EDTA, 1 µg RNase A per 1 OD cells were added to the lysed cells and incubated at 37 °C for 15 min. One volume of Roti^®^ Aqua phenol/chloroform/isoamyl alcohol (P/C/I, 25:24:1, pH 4.5–5) (Carl Roth GmbH + Co. KG, Karlsruhe, Germany) was added to the mixture and vortexed at full speed for 15 s. After incubating at 65 °C for 15 min, the tubes were centrifugated at room temperature for 15 min at 17,949 rcf. The aqueous upper phase was transferred to a fresh 2 mL-tube without disturbing the protein interphase or the organic lower phase. Ethanol precipitation was performed by adding 1/10 volume of 3 M sodium acetate, pH 5.2, and 2.5 volumes of 100% ethanol to the aqueous phase, followed by vortexing for 5 s. After precipitation for 1 h at −20 °C, dsRNA was pelleted at 17,949 rcf for 30 min at 4 °C. The dsRNA pellet was washed twice with 70% (*v*/*v*) cold ethanol, air dried, dissolved in 100 µL of nuclease-free water, incubated at 55 °C for 15 min and immediately placed on ice. For comparison with the original phenol–guanidine-based protocol, bacterial cells were also processed using TRIzol^TM^ (Invitrogen, Thermo Fischer Scientific, Waltham, MA, USA) as published in the literature [29]. Concentration and quality of purified dsRNA was assessed via absorbance measurement and gel electrophoresis, respectively.

### 4.4. Removal of High-Molecular-Weight Nucleic Acid Contamination by DNase Digestion

Removal of high-molecular-weight nucleic acid contamination from dsRNA obtained via the phenol–guanidine-based extraction with TRIzol^TM^ [29] was evaluated via DNase digestion. The final product from TRIzol^TM^ extraction was resuspended in nuclease-free water and DNase digestion was performed at room temperature for 15 min. A mock digestion was performed for comparison. Following DNase digestion, the nucleic acids were extracted with P/C/I (25:24:1, pH 7.5–8) and precipitated with ethanol. Concentration measurement and quality assessment via gel electrophoresis were performed as described above.

### 4.5. Cell Lysis Efficiency Testing

Efficiency of cell lysis by boiling HT115 (DE3) cells at 95 °C for two minutes after resuspension in 12.5 µL of SDS 0.1% (*w*/*v*) per OD_600_ of cells was evaluated through a cell viability assay. After boiling, cells were homogenized and spread into LB-agar 1.5% (*w*/*v*) plates containing ampicillin (100 µg/mL). As control, cells were resuspended in the same volume of sterile LB-medium 2% (*w*/*v*) and incubated at room temperature for two minutes. Then, cells were homogenized and aliquots of the undiluted cells suspension and of 1:100 dilution were spread into LB-agar plates. All plates were incubated overnight at 37 °C before evaluation of colony development. The experiments were performed in biological duplicates.

### 4.6. Assessment of pH and Serial P/C/I Extractions on dsRNA Purity

The protocol described in Section 4.3 was tested in four different modifications to further improve dsRNA purity: (1) cell lysis was performed at 95 °C for 2 min in 0.1% (*w*/*v*) SDS, 0.1 M sodium acetate, pH 4.5. The rest of the protocol remained unchanged. (2) the RNase A digest was performed in 300 mM sodium acetate, 10 mM Tris-HCl, 5 mM EDTA, pH 4.5. The rest of the protocol remained unchanged. (3) The reaction was acidified by addition of 2.5 volumes of 0.1 M sodium acetate, pH 4.5 before the addition of one-volume equivalent (1000 µL) of P/C/I (25:24:1, pH 4.5–5.0). The rest of the protocol remained unchanged. (4) The product from the first P/C/I extraction, obtained as described in 4.3 and resuspended in 100 µL of nuclease-free water, was mixed with 300 µL of either 0.1 M sodium acetate at pH 4.5 or 0.2 M sodium phosphate dibasic at pH 6.8 or 0.1 M sodium bicarbonate/sodium carbonate at pH 9.9. Then, a second extraction was performed using 400 µL of P/C/I (25:24:1, pH 4.5–5). The downstream steps of dsRNA ethanol precipitation, washing, and resuspension in 100 µL of nuclease-free water, were performed as described in Section 4.3. Concentration measurement and quality assessment via gel electrophoresis of the obtained dsRNA from each experiment were performed as described above.

### 4.7. Optimization of the Induction OD_600_

dsRNA was extracted from bacterial cells induced to produce dsRNA at OD_600_ of 0.4 or 0.8 using a phenol–guanidine-based protocol [29], two ethanol-based extraction protocols published in the literature [32,33], and the final version of the protocol developed in this study (two-step acidic P/C/I extraction, see Section 4.6). All extractions were performed as published with the exception of the phenol–guanidine-based protocol, where the RNase digestion was performed for 15 min instead of 5 min as originally published. Concentration measurement and quality assessment via gel electrophoresis were performed as described above. Extraction yields and dsRNA purity after induction at different cell densities was compared between all four protocols.

### 4.8. Systematic Comparison of dsRNA Extraction Protocol Efficiencies

To compare the yield and purity of phenol–guanidine-based [29], ethanol-based [32,33], and alkaline phenol-based [30,31] extraction protocols with the protocol developed in this study, dsRNA production in HT115 DE3 cells was induced at an OD_600_ of 0.4. Experiments were performed in three biological replicates and two technical replicates each. Importantly, the quantitative yields were calculated considering the initial amount of cells and volume of nuclease-free water in the final resuspension step of each protocol. The method by Ongavarrasopone et al. [29] was followed as published, except for an RNase A digestion time of 15 min instead of 5 min. The methods established by Posiri et al. [32] and Papic et al. [33] were followed in their entirety as published. The protocol established by Ahn et al. [31] was adapted by opening the cells via homogenization in tubes containing ceramic beads in a tissue homogenizer (Precellys^®^, Bertin Instruments, Montigny-le-Bretonneux, France) for two runs at 6000 rpm for 20 s with an intermediate incubation on ice for 1 min. The method established by Solis et al. [30] was followed in its entirety, with exception of using Monarch DNase I (1 U/µL) and Monarch DNase I reaction buffer (New England Biolabs, Inc., Ipswich, MA, USA) for DNase digestion instead of Turbo DNase and DNase buffer (Ambion/Life Technologies, Thermo Fischer Scientific Inc., Waltham, MA, USA). These results were compared to the final version of the protocol developed in this study: Bacterial cells were resuspended in 12.5 µL of 0.1 % (*v*/*v*) SDS per OD of cells and incubated at 95 °C for 2 min. Then, 16.25 µL of 300 mM sodium acetate, 10 mM Tris-Cl, 5 mM EDTA, pH 7.5, 1 µg of PureLink RNase A per OD of cells were added and incubated at 37 °C for 15 min. The equivalent volume of P/C/I (25:24:1, pH 4.5–5) was added, thoroughly mixed for 15 s and incubated at 65 °C for 15 min. After centrifugation at 17,949 rcf for 15 min at 20 °C, the aqueous phase containing dsRNA was carefully transferred to a new tube without disturbing the protein interphase and the organic lower phase. Ethanol precipitation was performed by adding 1/10 volume of 3 M sodium acetate, pH 5.2, and 2.5 volumes of 100% ethanol to the aqueous phase, followed by vortexing for 5 s and precipitation for 1 h at −20 °C. The dsRNA was pelleted at 17,949 rcf for 30 min at 4 °C and washed once with 70% (*v*/*v*) cold ethanol. The air-dried dsRNA pellet was dissolved in 100 µL of nuclease-free water and incubated at 55 °C for 10 min. Optional DNase digestion was performed with 25 µL DNase I mix and incubated at 30 °C for 15 min. Then, 375 µL of 0.1 M acetate buffer at pH 4.5 was added before repeating the P/C/I extraction and ethanol precipitation as described above, this time applying an additional ethanol wash step. Finally, the dsRNA pellet was dissolved in 100 µL of nuclease-free water, incubated at 55 °C for 10 min, and immediately placed on ice. DsRNA yields were calculated as total amount of nucleic acid quantified via spectrophotometry at 260 nm divided by the total OD of bacterial cells used for extraction to result in µg dsRNA/1 OD of cells. The quality of extracted dsRNA was inspected via gel electrophoresis analyzing the same volume of extracted dsRNA from each technical and biological replicates from each extraction method.

### 4.9. Analysis of Band Intensity from Gel Electrophoresis

Images acquired from the gel documentation system were analyzed using the software Adobe Photoshop 2023 (Adobe Inc., 2023, San José, CA, USA). In general, the bands or the region of interest or the complete lane were identified and their integrated density was quantified.

### 4.10. Statistical Analysis

Data analysis was carried out using the software SigmaPlot (Version 14.0, Systat Software Inc., San José, CA, USA) and MiniTab^®^ (Minitab, LLC., State College, CA, USA). Yields of nucleic acids extracted from bacterial cells induced to produce dsRNA at OD_600_ of 0.4 or 0.8, and yield of extracted dsRNA using different published methods and the optimized version of our protocol were analyzed via one-way analysis of variance (ANOVA), with Shapiro–Wilk test for normality and Brown–Forsythe test for equal variance. Holm–Sidak method was used for multiple comparison of the data from each condition, when the differences in the mean values among the groups were greater than would be expected by chance (α = 0.05). Data of the relative dsRNA band intensity, which failed Shapiro–Wilk test for normality, underwent a Box Cox transformation [39] prior to conducting the ANOVA. For the comparative analysis of the amounts of nucleic acids extracted using different published methods and the optimized version of our protocol, the yields of nucleic acid per OD_600_ for each extraction method were analyzed via Welch’s test, which does not assume equal variances for the analysis. The means were compared with the Games–Howell pairwise comparison at 95% confidence level.

## Figures and Tables

**Table 1:** dsRNA purification costs based on the organic reagent costs for each protocol used in this study. Prices of the organic reagent used for calculations are list prices for the largest commercially available packing size at the time of writing this manuscript.

Protocol	Required Volume of Organic Reagent Per OD Cells (mL)	Price of Organic Reagent (EUR/mL)	Absolute dsRNA Yield ^1^ (µg/OD)	Organic Reagent Costs for 400 mL Culture (EUR)	Organic Reagent Costs Per mg of Purified dsRNA (EUR)
Acid phenol	0.0688	0.16	3.87	3.58	2.90
Phenol–guanidine	0.2000	2.14	2.86	135.93	149.57
Ethanol I	0.0375	0.08	1.47	0.93	1.99
Ethanol II	0.0350	0.08	095	0.87	2.86
Alkaline phenol I	0.0788	0.27	0.71	6.63	29.27
Alkaline phenol II	0.0627	0.27	0.58	5.28	28.32

^1^ Absolute dsRNA yields were calculated based on the integrated density of the dsRNA band compared to the integrated density of the whole lane quantified by Adobe Photoshop (Adobe Inc., 2023, San José, CA, USA) and the nucleic acid yield as determined by spectrophotometric analysis (see Figure 6).

## Data Availability

All data created in this study is included in the manuscript.
